# A Global Scoping Review of Clinicians’ Perceptions of Anorectal Biofeedback Compared with Novel Australian Data

**DOI:** 10.3390/jcm15020835

**Published:** 2026-01-20

**Authors:** Minha Lee, Vincent Ho, Jerry Zhou

**Affiliations:** 1 School of Medicine, Western Sydney University, Campbelltown, NSW 2560, Australia; 2 Department of Gastroenterology, Campbelltown Hospital, Campbelltown, NSW 2560, Australia

**Keywords:** biofeedback, anorectal disorder, anorectal disorders, faecal incontinence, constipation, attitude of health personnel, health knowledge, attitudes, practice, Australia, clinical practice pattern, clinical practice patterns

## Abstract

**Background/Objectives**: Biofeedback therapy is a technique that trains individuals to change their physiological activity for the purpose of improving their health. Despite its proven efficacy in 70 to 80% of patients in clinical trials, biofeedback is significantly underutilised in clinical practice worldwide. This scoping review aims to synthesise the current evidence on clinicians’ attitudes, knowledge, and experiences with anorectal biofeedback, highlight gaps in the existing literature, and guide future research directions. These findings are compared with new local Australian data. **Methods**: Systematic searches were conducted on five electronic databases including MEDLINE, Embase, CINAHL, Scopus, and APA PsycInfo. Eight articles were retrieved from title and abstract, full text, and reference list screening using Covidence. **Results**: The scoping review revealed substantial heterogeneity in the clinical indications for biofeedback. Both the scoping review and Australian study reported that over half of clinicians could not accurately define biofeedback or report familiarity with the technique, and that barriers to the implementation of biofeedback included long wait times, lack of trained personnel, and limited access to equipment. In the Australian study, proposed ways to improve the uptake of biofeedback included education of health professionals, government funding, and increased access to facilities. **Conclusions**: This scoping review and Australian cross-sectional study demonstrate that clinicians have limited knowledge of biofeedback and that a complex interplay between systemic barriers to access hinder its implementation. Further research into clinicians’ attitudes towards biofeedback should be conducted in more countries to build a more robust body of evidence.

## 1. Introduction

Biofeedback therapy is a technique that trains individuals to change their physiological activity for the purposes of improving their health [1]. Biofeedback was first described in the literature as a potential treatment for anorectal disorders such as faecal incontinence in 1974 [2]. During the late twentieth and early twenty-first centuries, biofeedback remained a novel option for the short-term treatment of anorectal disorders, improving symptoms in over half of patients [3,4].

Biofeedback is currently recognised as a safe and effective treatment for anorectal disorders in the official clinical guidelines of countries such as Australia [5], Mexico [6], the United States, and the United Kingdom [7]. In 2010, the Australian Prescriber published an article stating that biofeedback was effective in treating defecatory disorders in up to 75% of cases [5]. The 2011 Mexican guidelines on the treatment of faecal incontinence strongly recommended biofeedback therapy for individuals who are unresponsive to conservative medical treatment [8]. In 2015, the American Neurogastroenterology and Motility Society (ANMS) and the European Society of Neurogastroenterology and Motility (ESNM) published a joint position paper that recommended biofeedback for the short-term and long-term treatment of constipation with dyssynergic defecation and faecal incontinence [7]. The position paper further highlighted the potential efficacy of biofeedback for the short-term treatment of levator ani syndrome and solitary rectal ulcer syndrome with dyssynergic defecation. Clinical trials have shown that biofeedback effectively treats 70% to 80% of patients with dyssynergic defecation and 76% of patients with refractory faecal incontinence [9].

Despite the proven efficacy of biofeedback for anorectal disorders, only about 1.1% of Australians with bowel control problems underwent anorectal biofeedback manometry therapy in 2024 [10]. In the United States, biofeedback accounted for less than 3.1% of functional bowel disorder treatments in 2021 [11]. Studies have proposed potential reasons for the low uptake of biofeedback such as lack of education and training of healthcare providers [7,8], lack of insurance coverage [12], and limited facilities providing the therapy [8,13,14,15]. However, to the best of our knowledge, there is currently no study that comprehensively synthesises the global literature on clinicians’ knowledge, perceptions, and experiences with anorectal biofeedback.

This scoping review aims to synthesise the current evidence on clinicians’ perceptions of biofeedback, highlight existing gaps in the literature, and inform future research into anorectal biofeedback. These findings are compared with novel Australian data from a survey-based, cross-sectional study that investigates the perspectives of gastroenterologists in Sydney, New South Wales. Understanding the attitudes of clinicians such as gastroenterologists, colorectal surgeons, physiotherapists, and other relevant health professionals is critical to determine the current applications, accessibility, and efficacy of anorectal biofeedback. These findings could assist governing bodies and policymakers to develop initiatives aimed at improving the access and delivery of biofeedback therapy.

## 2. Methods

### 2.1. Scoping Review

#### 2.1.1. Study Design

This scoping review conforms to the guidelines outlined in the Preferred Reporting Items for Systematic Review and Meta-analyses–Scoping Review (PRISMA-ScR) statement (Supplementary Table S1). A study protocol was not registered in a public registry.

#### 2.1.2. Ethical Considerations

Ethics approval was not required for this scoping review, as the study involved analysis of publicly available literature. The included unpublished study was reviewed and exempted from a full ethics application by Western Sydney University Human Ethics Committee.

#### 2.1.3. Search Strategy

Systematic searches were conducted on five electronic databases including MEDLINE, Embase, CINAHL (Ebsco), Scopus, and APA PsycInfo (Ebsco) in May 2025. The search was based on four main concepts: (1) clinicians involved in the diagnosis, referral or management of anorectal disorders, (2) biofeedback, (3) anorectal disorders, and (4) attitudes, knowledge and/or experiences of clinicians (see Appendix A for the full search strategies). Articles were retrieved based on keywords in their title and abstract. To identify additional potential articles, the reference lists of eligible articles were manually searched. Selected articles were retrieved using Covidence, which facilitated reference management and the removal of duplicate entries.

#### 2.1.4. Eligibility Criteria

Peer-reviewed studies that investigated the four main concepts were included. No date or language restrictions were applied to ensure comprehensive coverage of all relevant literature given the limited number of studies available on this topic. Clinical trials, reviews, editorials, conference abstracts, book chapters, protocols, and animal trials were excluded. Studies investigating the perceptions of patients, students, or children, rather than clinicians, were also excluded.

#### 2.1.5. Study Selection

The study selection process was carried out using Covidence, starting with the importation of all eligible studies to remove duplicates. Two authors (M.L. and J.Z.) independently screened the titles and abstracts to determine eligibility based on the established inclusion and exclusion criteria. Subsequently, the full texts of eligible articles were reviewed by the same authors for further evaluation. Any disagreements were resolved by a third researcher (V.H.).

#### 2.1.6. Data Extraction

M.L. and J.Z. extracted data from the included articles using a standardised table format in Excel, which included the following information: lead author’s name, year of publication, country, study design, data collection method, anorectal condition, monetary incentive, analysis approach, sampling frame, sample size, response rate, participant occupation(s), age and gender, practice setting, specialty training/experience, knowledge/understanding of biofeedback, equipment and method used in biofeedback, experience with biofeedback/frequency of anorectal condition, training/supervision of biofeedback, perceived effectiveness of biofeedback, overall attitudes towards biofeedback, access to biofeedback, barriers to biofeedback use, and ways to improve biofeedback uptake. The characteristics and findings of the included studies are summarised in Table 1. Discrepancies between reviewers were resolved through mutual consensus. 

#### 2.1.7. Risk of Bias Assessment

Risk of bias was assessed by two authors (M.L. and J.Z.) independently using the Joanna Briggs Institute (JBI) checklist for analytical cross-sectional studies (see Appendix B), which consists of eight items rated as ‘yes’, ‘no’, ‘unclear’, or ‘not applicable’. Disagreements raised during the risk of bias assessment were resolved by discussion with the third author (V.H.). Since the included studies assessed perceptions of clinicians, items 3 and 4 relating to measurement of the exposure and condition were not applicable and were excluded from the overall bias assessment. Item 7 was interpreted to include consideration of how low response-rates and convenience samples could lead to potential bias and reduce external validity. Studies that received ‘yes’ answers to five or six of the six questions were considered low risk of bias, three or four were considered moderate risk, and one or two were considered high risk.

### 2.2. Australian Cross-Sectional Study

#### 2.2.1. Description

This section reports on an unpublished primary study not included in the scoping review. This Australian cross-sectional study aims to determine the perceptions of Sydney-based gastroenterologists on anorectal biofeedback with respect to its definition, application, and efficacy, and to identify barriers to its uptake.

#### 2.2.2. Rationale for Inclusion

This study was included to address a clear knowledge gap identified by the scoping review. While the review summarises the international literature, there is no published data describing Australian clinicians’ familiarity with or access to anorectal biofeedback. The Australian study therefore provides the necessary local context to interpret the applicability of the international findings within a different healthcare system. To avoid methodological bias, this study was not included in the scoping review dataset and is interpreted descriptively as complementary, exploratory evidence.

#### 2.2.3. Ethical Considerations

The research received an exemption from ethics approval based on low risk by the Western Sydney University Human Research Ethics Committee (Ethics Reference EX-H16126) on 1 July 2024.

#### 2.2.4. Participants

The study involved completing an online survey, which was developed using Qualtrics. First, searches of every gastroenterologist and gastroenterology clinic in Sydney were made on HealthDirect Australia and Whitecoat using all Sydney postcodes. Sydney was selected as a pragmatic sampling frame because it contains a larger concentration of tertiary centres and anorectal physiology centres compared to other regions. From this search, a list of 237 gastroenterologists and their contact details (email address or phone number) was created, of which 223 were contactable. The online survey and participant information sheet were emailed to the gastroenterologists whose email address was available online. Additional phone calls were made to gastroenterology clinics requesting that the online survey and participant information sheet be forwarded to the designated gastroenterologists. Follow-up emails were sent one and two weeks after the initial email. Informed consent was provided by pressing ‘Yes’ in response to the question ‘Please click ‘Yes’ if you would like to proceed or ‘No’ if you would like to opt out’. Each gastroenterologist was asked to complete the survey based on their personal knowledge, attitudes, and experiences with anorectal biofeedback. Forty six gastroenterologists consented to and completed the online survey (response rate of 20.6%).

#### 2.2.5. Survey Design

The survey was developed using Qualtrics by two investigators: a clinical researcher who specialises in gastrointestinal physiology with over 10 years of translational research experience and a medical student with experience in anorectal biofeedback. The draft survey consisted of 7 sections including the following: (i) background (1 question), (ii) eligibility (2 questions), (iii) demographics (6 questions), (iv) knowledge about biofeedback (2 questions), (v) experience with anorectal biofeedback (8 questions) or anorectal conditions (4 questions), (vi) biofeedback training (5 questions), and (vii) attitudes towards biofeedback (4 questions). See Supplementary Materials S2 for the full survey.

The content validity of the draft survey was evaluated by the Lawshe Technique with a pilot study in which 4 gastroenterologists, who were blinded to the process of creating the draft survey, were surveyed to assess the content validity of the draft survey. Each gastroenterologist was asked to provide their opinion on whether each survey question was ‘necessary’, ‘necessary but not sufficient’, or ‘not necessary’. According to the recommendations of the validation group, 2 questions in section (iii) and the entirety of section (vi) were removed after being deemed ‘not necessary’. The wordings and answer options to several questions were amended in response to constructive feedback from the gastroenterologists. After the pilot study, the final version of the survey included 6 sections with a total of 17 or 21 questions depending on the response in section (iv).

The first section provided the participants with background information about the study and the option to consent to the survey. The second section verified the 2 eligibility criteria for participation in this study: (1) gastroenterologist who has undertaken specialty training in Australia and (2) has worked in Australia for the past 3 years. The demographic data of the clinicians in the third section included gender, years of practice in gastroenterology, type of practice, and local health district(s) they have worked in.

The fourth section examined gastroenterologists’ knowledge of biofeedback through 2 questions, one of which requested a short response answer and the other asked them to select ‘yes’ or ‘no’. In the fifth section, depending on whether the gastroenterologist was aware of anorectal biofeedback, a combination of short response and multi-choice questions assessed either their understanding of the therapy or approach to treating anorectal conditions. In the sixth section, gastroenterologists’ attitudes towards the efficacy of anorectal biofeedback were gauged using a five-point Likert scale between 1 (not effective at all) and 5 (extremely effective).

#### 2.2.6. Statistical Analysis

Statistical analysis was performed by using Microsoft Excel (Microsoft Corporation, USA) and SPSS Version 29 (SPSS IBM, New York, NY, USA) for Windows Version 11. Descriptive statistics were calculated (number and percentage for qualitative data and number, percentage, skew number, median, range, minimum and maximum values for quantitative data). Chi-square analysis was conducted to examine the statistical significance of relationships between variables. The level of statistical significance was set at 5%.

To correctly define anorectal biofeedback in question 1 of section (v), gastroenterologists needed to cover both its aim as per the definition ‘sensory and muscular retraining of the rectum and pelvic floor with the goals of improving sensation, muscular relaxation or strengthening, and improving the defecation dynamics’ [24] and its process as seen in the definitions ‘connection to sensors that receive feedback information about the body’ [25], ‘variety of systems designed to give patients direct, visual feedback about internal psychophysiological processes’ [26] and ‘develop an awareness of more subtle interoceptive physiologic phenomena so that they come under direct voluntary control by the patient’ [26]. The suitability of each definition was manually assessed by a researcher (M.L.).

## 3. Results

### 3.1. Scoping Review

#### 3.1.1. Study Selection

**Figure 1:**
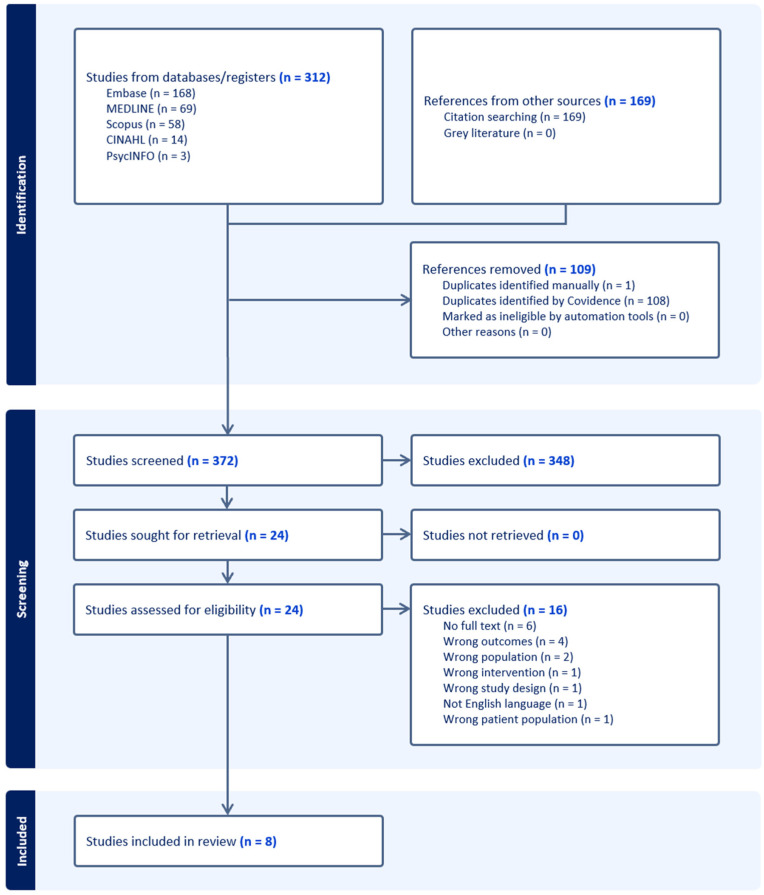
PRISMA diagram reflecting article review process.

The initial database searches retrieved 312 studies and later citation searches retrieved 169 studies, of which 109 duplicates were removed and an additional 348 were excluded based on title and abstract review. This left 24 studies for full text review, 8 of which were included in the final review. The details of the study selection process are outlined in the PRISMA flowchart (Figure 1).

#### 3.1.2. Risk of Bias

Five of the eight assessed studies were classified as moderate-risk and three as high-risk. Most studies clearly defined the inclusion criteria and described the subjects and settings in detail. However, few studies identified confounding factors, and no studies stated strategies to deal with the confounding factors. The majority of the studies had low response rates below 30% or did not provide a response rate, reducing external validity and increasing the risk of non-response bias. Half of the studies provided sufficient detail regarding the statistical analyses used.

#### 3.1.3. Description of Study Characteristics

The eight included studies were survey-based, cross-sectional studies that employed descriptive statistics, univariate analysis, and/or thematic analysis. The countries in which the studies were conducted included Australia (n = 2), New Zealand (n = 2), USA (n = 2), UK (n = 1), Italy (n = 1), France (n = 1), and Spain (n = 1). The sample size of the studies ranged between 37 and 484, with a mean of 185. Six of eight studies reported the response rate, which ranged between 5.80% to 68.50% and was 27.23% on average. This is significantly lower than the minimum acceptable response rate of 60% [27,28].

#### 3.1.4. Description of Participant Characteristics

The studies most commonly surveyed colorectal surgeons (5/8 studies), gastroenterologists (2/8 studies), and proctologists (2/8 studies). General surgeons, gynaecologists, urologists, nurses, physiotherapists, clinical physiologists, and other physicians were also surveyed. Three studies [17,18,21] reported the age of participants, of which one found that the majority of colorectal surgeons were aged 40 to 60 years [17], another found a mean of 52 years for gastroenterologists and 51 years for colorectal surgeons [18], and a third found a mean of 51.7 years for colorectal surgeons and proctologists [21]. Two studies [20] had more male than female participants (55.2% vs. 40.5% [20] in one study and 67% vs. 33% in another study). Participants worked in a range of clinical settings, including public and private hospitals in either metropolitan or rural regions, inpatient or outpatient settings, domestic or overseas, and university affiliated hospitals. In two studies [17,23], the location of current work and specialty training of participants was diverse, including Australia, New Zealand, UK, and USA among other countries. Three studies [18,20] reported the clinicians’ years of practice, which ranged from 0 to 45 years.

#### 3.1.5. Knowledge of Biofeedback

Two studies [16,22] assessed clinicians’ knowledge and understanding of biofeedback. One study [16] found that the majority of clinicians (63%) could not provide an appropriate definition of biofeedback, and the other study [22] found that most clinicians (62%) had little knowledge of biofeedback.

#### 3.1.6. Clinical Applications of Biofeedback

The included studies identified that biofeedback is used as a treatment for obstructed defecation, low anterior resection syndrome (LARS), chronic idiopathic constipation, pelvic floor disease, solitary rectal ulcer syndrome (SRUS), faecal incontinence, anal pain, post-surgical rehabilitation, and tenesmus. N.A. Coppersmith et.al. [20] reported 13.5% of colorectal surgeons treated LARS with biofeedback, less frequently than lifestyle modifications with drugs (32.7%), physical therapy (18.5%), and only lifestyle modifications (16.5%). M.L. Weinman et.al. [22] found that 23.2% of physicians felt that biofeedback was not indicated in the treatment of faecal incontinence, with only a minority (4.2%) reporting that they would use biofeedback as the primary treatment. L.M. Jimenez-Gomez et.al. [23] found that 8.3% of American surgeons and 10.0% of Spanish surgeons preferred biofeedback to treat LARS. This proportion was lower than lifestyle and dietary management with drugs (48.8% of American surgeons and 40.7% of Spanish surgeons) and without drugs (12.8% of American surgeons and 41.3% of Spanish surgeons).

#### 3.1.7. Method and Equipment Used in Biofeedback

One study [16] reported that biofeedback therapy involved lifestyle, dietary and medication advice, exercises such as urge resistance and brace pump technique, toileting position, and transanal irrigation. They also identified the use of equipment such as visual, muscle stimulation and auditory devices. Concurrent rehabilitation modalities such as muscle stimulation, electrostimulation, and physiokinesitherapy were sometimes employed alongside standard biofeedback [16,19].

#### 3.1.8. Training and Supervision of Biofeedback

A study conducted by K.J. Etherson et.al. [16] reported that a greater proportion of clinicians underwent informal training for biofeedback compared with formal, assessed training courses (95% vs. 65%). Informal training was most commonly delivered by peers (59%) and mentors (46%), with a minority being carried out by manufacturers of biofeedback equipment (5%). Over half of clinicians who reported informal training were self-taught, using journal articles, peer observation, books, conferences, and study days among other methods. Formal supervision of biofeedback was most often carried out by senior clinical colleagues and peers of similar grade (38% reported each). A similar trend was seen in reports of informal supervision (30% and 41%, respectively). Less than half of clinicians received regular supervision and 26% received no supervision.

#### 3.1.9. Perceived Effectiveness of Biofeedback

The reported efficacy of biofeedback in treating anorectal disorders varied between studies. K.J. Etherson et.al. [16] reported that nurses and physiotherapists in Southern UK perceived biofeedback to be more effective than their counterparts in Northern UK (mean efficacy of 70% vs. 46%). C. Gouriou et.al. [21] found that the majority (68%) of proctologists and colorectal surgeons believed that biofeedback therapy should be proposed for the treatment of SURS prior to surgery. L.M. Jimenez-Gomez et.al. [23] discovered that 14.2% of American surgeons and 23.3% of Spanish surgeons reported biofeedback as the most effective treatment for defecatory functional impairment after LARS, with drug and dietary treatment being the most common modality (55.6% of American surgeons and 40.7% of Spanish surgeons).

#### 3.1.10. Level of Access to Biofeedback

Two studies [17,18] reported that over half of clinicians had access to biofeedback therapy (69% of colorectal surgeons in one study [17], 73% of gastroenterologists and 82% of colorectal surgeons in another study [18]). M.L. Weinman et.al. [22] reported that more physicians stated that biofeedback was covered by health insurance than those who did not (27% vs. 16.9%), with 47.1% being undecided. L. Losacco et.al. [19] found that the majority (86.5%) of general surgeons, gynaecologists, and urologists in Italy reported that hospitals did not have a dedicated performance code for pelvic multidisciplinary examinations, which involve biofeedback.

#### 3.1.11. Barriers to Use of Biofeedback

One reference [16] identified that barriers to biofeedback usage include long wait times and lack of qualified personnel or equipment. N.A. Coppersmith et.al. [20] reported that an increased number of years in practice of colorectal surgeons was associated with a reduced odds of using biofeedback (OR = 0.74, 95% CI = 0.54–1.00, *p* = 0.050).

### 3.2. Australian Cross-Sectional Study

#### 3.2.1. Study Population

**Figure 2:**
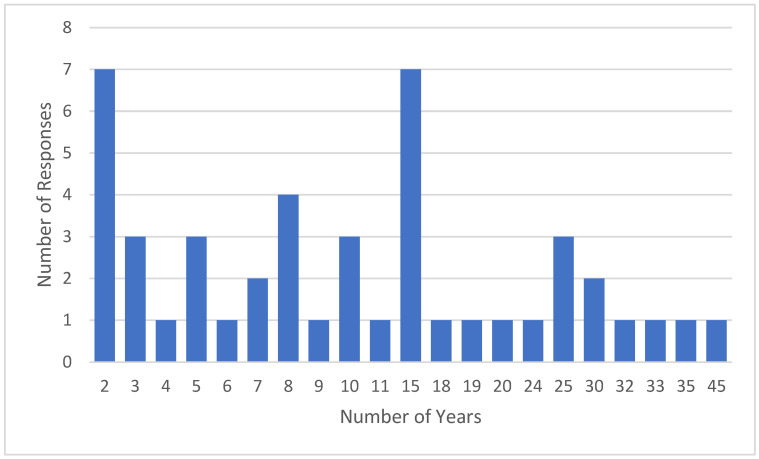
Years of experience practising gastroenterology (n = 46).

The characteristics of the 46 gastroenterologists included in this study are detailed in Table 2. The cohort is comprised of 31 males (67%) and 15 females (33%); 72% of the gastroenterologists practise in both public and private settings, while 15% practise only in private settings and 13% only in public settings. New South Wales (NSW) Health is the public health system within the state of NSW, Australia and is comprised of 15 local health districts (LHDs), 6 of which are in Sydney. The distribution of LHDs across Sydney in which the gastroenterologists previously or currently worked is outlined in Table 3. The cohort had experience working in all the six LHDs across Sydney, with 26% in the Sydney LHD, 23% in the South Western Sydney LHD, 17% in the South Eastern Sydney LHD, and 15% in the Northern Sydney LHD (Table 3). The median experience practising gastroenterology was 9.5 years (range: 2–45 years, skew: 1.03) (Figure 2).

#### 3.2.2. Perceptions of Anorectal Biofeedback

**Figure 3:**
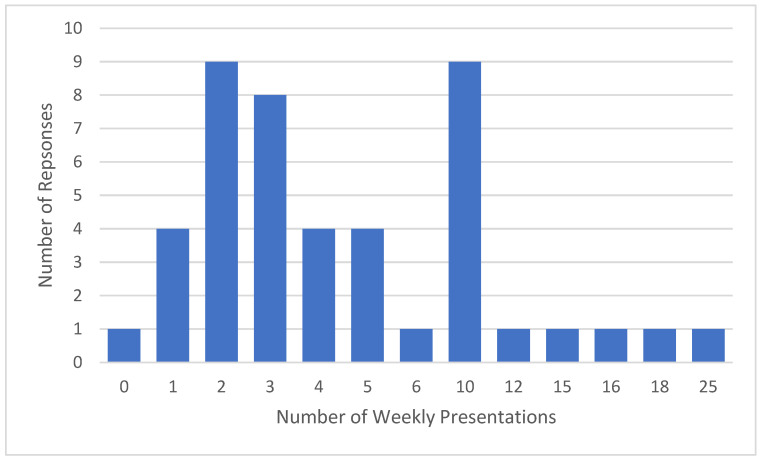
Frequency of patient presentations of bowel symptoms relating to anorectal dysfunction per week (n = 46).

Gastroenterologists reported seeing a median of four patients with bowel symptoms relating to anorectal dysfunction per week (range: 0–25, skew: 1.60) (Figure 3).

Of the 46 gastroenterologists, 44 (96%) reported that they were aware of anorectal biofeedback; of these, only 21 (48%) could provide an appropriate definition of anorectal biofeedback. When asked how the usage of anorectal biofeedback could be improved, four of the choices were cited by 10% or more of the gastroenterologists: 20% chose education of gastroenterologists, 19% chose wider access, 12% chose government subsidies, and 11% chose education of general practitioners (Table 3).

#### 3.2.3. Experiences with Anorectal Biofeedback

Of the 44 gastroenterologists aware of anorectal biofeedback, 25 (57%) reported that the therapy was offered as a treatment at their clinic or hospital. When the remaining 19 gastroenterologists were asked why the therapy was not offered, 12 (39%) indicated that there was no one qualified to deliver it, 8 (26%) indicated that costs were a barrier, and 6 (19%) indicated that lack of knowledge of biofeedback was an issue (Table 4).

**Figure 4:**
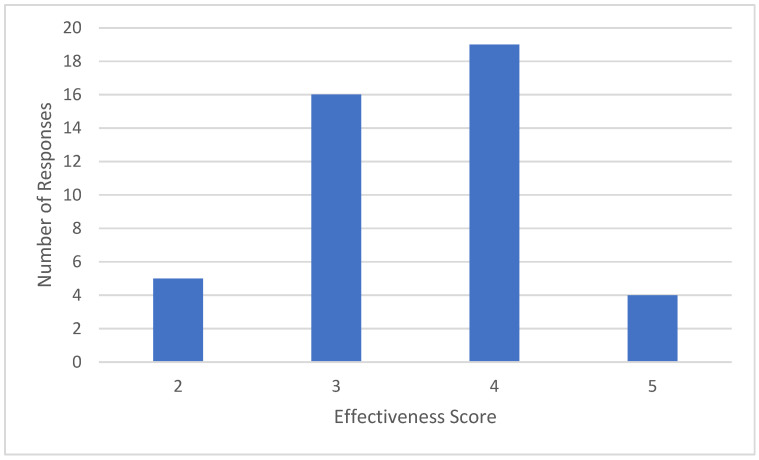
Effectiveness of anorectal biofeedback therapy (n = 46).

Gastroenterologists were asked to rate the effectiveness of anorectal biofeedback on a scale of 1 to 5, where 1 is ‘no effect’ or ‘produced harm’ and 5 is ‘cured’ or ‘significant improvement’. Most gastroenterologists (n = 35) believed that anorectal biofeedback had moderate effectiveness (3 or 4 out of 5), with few (n = 4) indicating biofeedback resulted in significant improvement and none indicating biofeedback had no effect (Figure 4).

The cohort reported that a median of 50% of patients benefitted from anorectal biofeedback therapy (range: 2–80%), although 17% of gastroenterologists indicated that they were unsure that there was any benefit. The effectiveness of anorectal biofeedback was gauged from direct patient feedback (70%), feedback from a nurse or physiotherapist (13%), objective parameters (13%), and feedback from a colorectal surgeon (3%).

In response to a question inquiring into the conditions treated by anorectal biofeedback, 19% chose faecal incontinence, 18% chose dyssynergic defecation, 17% chose chronic constipation, and 17% chose anorectal dyssynergia (Table 5). When questioned about the components of anorectal biofeedback therapy, 17% reported lifestyle advice, 15% toileting position, 14% dietary advice, 13% medications advice, and 12% exercises (Table 6). Among respondents, 55% were unsure about the equipment used in anorectal biofeedback therapy, 26% nominated visual devices, 13% nominated muscle stimulation devices, and the remaining 6% nominated auditory devices.

Of the two gastroenterologists who were unaware of anorectal biofeedback, one reported only using dietary advice to treat faecal incontinence. When asked about their treatment of chronic constipation, one of the two used osmotic or stimulant laxatives, and one used toileting position advice.

## 4. Discussion

### 4.1. Scoping Review

The safety and efficacy of biofeedback therapy for the nonpharmacological treatment of anorectal disorders such as constipation with dyssynergic defecation and faecal incontinence is well established in the literature [3,4,5,7,8,9]. Despite the proven benefits of anorectal biofeedback therapy, its global uptake has been historically low [10,11] due to factors such as lack of education, insurance, and facilities [7,8,12,13,14,15]. This scoping review provides the first comprehensive synthesis of clinicians’ knowledge, perceptions, and experiences of anorectal biofeedback therapy across multiple countries and health professions to better understand the reasons for its underutilisation in clinical practice.

In this scoping review, a significant proportion of clinicians demonstrated limited knowledge of anorectal biofeedback, with over half of clinicians being unable to accurately define the therapy or report familiarity with the technique. This finding contrasts with the endorsement of biofeedback in international consensus statements and clinical guidelines [5,7,8], highlighting the disconnect between evidence-based recommendations and clinician awareness of biofeedback.

There was substantial heterogeneity in the clinical indications for which biofeedback therapy was considered. Although obstructed defecation and faecal incontinence were the most commonly cited conditions, some clinicians reported applications in treating other anorectal disorders such LARS, SRUS, and pelvic floor rehabilitation. This breadth underscores the lack of standardisation in the role of biofeedback in treating anorectal conditions across different specialties and countries, perpetuating knowledge gaps and lack of confidence among health professionals in recommending or delivering biofeedback.

Health insurance coverage of biofeedback therapy varies globally. In the US, the public Medicare system offers two CPT (Current Procedural Terminology) codes that reimburse patients for biofeedback training of the perineal muscles, anorectal or urethral sphincter [29]. The Italian National Health Service (SSN) reimburses anorectal biofeedback therapy as a form of pelvic floor rehabilitation via an ICD-9-CM code [30]. Despite the availability of reimbursement for biofeedback in the US and Italy, a majority of surveyed American and Italian clinicians reported no health insurance coverage for biofeedback or were undecided about it. Additionally, biofeedback accounted for less than 3.1% of functional bowel disorder treatments in the US in 2021 [11]. Thus, even with the availability of financial subsidies, lack of awareness of these supports significantly impedes the use of biofeedback.

Another factor contributing to underutilisation and reduced efficacy of anorectal biofeedback, which was not mentioned in the included studies, is poor patient adherence to the therapy. According to a 2018 study, completion of the therapy and adherence to the homework are major determinants of the success of biofeedback treatment. Long treatment courses, transport and work commitments, and embarrassment from the content is associated with decreased patient compliance [31]. Y. Mazor et. al. compared an abbreviated biofeedback program involving fewer in-person, instrumented sessions and supplementary phone calls between visits against a standard protocol [32]. The study found that, although patient satisfaction was slightly higher with full-length biofeedback, both groups had similar magnitudes of symptom and quality of life improvement. Thus, a compressed or intensive biofeedback regime is an effective option for select patients travelling from interstate or rural areas.

Research has investigated the efficacy of home or hybrid biofeedback programs in improving the availability and use of the treatment. Studies reported that home or hybrid biofeedback was not inferior to clinic biofeedback in the treatment of dyssynergic defecation and faecal incontinence. However, widespread adoption of remote biofeedback has been limited by its inability to monitor compliance or exercise quality. To solve this issue, Zhou et. al. proposed an Internet of Medical Things (IoMT) system, which involves a sensing probe, smartphone app, and cloud-based portal to allow clinicians to monitor patient progress remotely [33]. A 2024 RCT demonstrated that the IoMT device was not inferior to conventional biofeedback for symptom improvement and bowel control satisfaction [34]. Hence, remote programs could help mitigate geography-related underutilisation of biofeedback.

### 4.2. Comparison to Australian Cross-Sectional Study

This cross-sectional study is the first to report on Australian gastroenterologists’ perceptions anorectal biofeedback in terms of its definition, application, and efficacy, as well as reasons for the limited use of biofeedback therapy. The study provides a local perspective from metropolitan Australian clinicians against which the international findings of the scoping review can be compared.

The Australian study mirrored the international literature’s reports of low clinical uptake of anorectal biofeedback despite clinicians having access to the therapy. However, unlike the US and Italy, which provide government subsidies for anorectal biofeedback, Australia lacks an MBS (Medicare Benefits Schedule) item number for biofeedback therapy [35], resulting in a substantial financial burden for most patients. In this study, most Australian gastroenterologists reported having access to biofeedback but national data showed that only around 1.1% of Australians with bowel control problems underwent anorectal biofeedback manometry therapy in 2024 [10]. These statistics indicate that the physical availability of biofeedback services is insufficient to encourage widespread uptake without the necessary financial support.

Furthermore, the Australian study reinforces the scoping review’s findings that clinicians have limited awareness and knowledge of anorectal biofeedback. The study found that the majority of gastroenterologists could not accurately define biofeedback, which aligns with the results of two studies in the scoping review [16,22]. This similarity highlights that lack of clinician awareness of biofeedback is common to both local and international contexts and may be a contributing factor to its low clinical use.

There are similarities and differences in the approach used for biofeedback reported by the scoping review and our Australian study. The most commonly used equipment in the UK was digital or auditory devices [16] compared with visual devices in Australia. In both countries, the most frequently used methods included lifestyle and dietary advice, toileting position, and exercises. These results show that, although there is consistency in the approach for biofeedback, the exact method of delivery may differ depending on factors such as the country’s guidelines or conventions, clinician’s preferences, and availability of equipment. Further research should be conducted into the differences in the efficacy of biofeedback based on the equipment and method employed to enable clinicians to maximise the effectiveness of the therapy.

The Australian study reported similar barriers to the implementation of biofeedback as the scoping review, such as long wait times, lack of trained personnel, and limited access to equipment. Another reason for the low uptake of biofeedback in clinical practice is its labour intensive nature, which may cause patients to lose motivation to comply with the treatment over time. Factors such as long-distance travel, lack of time, and high costs contribute to reduced patient adherence to biofeedback in the real world, outside of the highly controlled conditions of clinical trials.

### 4.3. Recommendations

The results of this scoping review and Australian cross-sectional study have several important implications for clinical practice, health policy, and future research. Increasing formal training opportunities for gastroenterologists, colorectal surgeons, physiotherapists, and nurses could improve their familiarity with biofeedback and increase clinical implementation. The development of a globally or nationally standardised protocol would make it easier to teach clinicians about biofeedback and scale the therapy [36].

Systemic barriers such as insurance coverage and limited facilities should be addressed by the relevant governing bodies by establishing billing codes for anorectal biofeedback and funding more tertiary biofeedback centres. Alternative options such as abbreviated regimes and home programs should be explored by clinicians to maximise patient adherence to the therapy. Other strategies to improve compliance include dedicated education sessions to improve patient buy-in, bowel diaries and goal setting to increase engagement, and scheduled follow-up sessions to sustain behavioural changes [36].

Future research should explore clinicians’ attitudes towards biofeedback in different countries and with diverse health professions to build a more substantial body of evidence from which more robust conclusions can be derived. Qualitative approaches may be particularly valuable to capture nuanced professional experiences and institutional challenges related to biofeedback therapy. Studies could also evaluate interventions aimed at improving knowledge, confidence, and uptake of biofeedback among clinicians.

### 4.4. Strengths and Limitations

#### 4.4.1. Scoping Review

A key strength of this scoping review is its systematic and comprehensive search across five databases, supplemented by citation tracking and the inclusion of relevant, unpublished data, which enhance the breadth of the evidence. The use of a structured extraction framework and approach enabled consistent synthesis across diverse studies. It is the first study to synthesise all the global evidence on clinicians’ views and experiences of anorectal biofeedback, guiding future research directions.

However, a limitation of this review is its reliance on cross-sectional, survey-based studies with modest response rates, which are subject to response bias and may not be representative of the clinicians involved in anorectal biofeedback therapy. Most studies did not address confounding factors or explore the differences between specialties in depth. Although some studies used validated survey instruments, others relied on self-developed tools without formal validation. These methodological issues underscore the need for more rigorous, mixed-methods research into anorectal biofeedback.

#### 4.4.2. Australian Cross-Sectional Study

A strength of the study is that it is the first to investigate Australian gastroenterologists’ perceptions of anorectal biofeedback and the reasons for the limited use of the therapy, filling this gap in the literature. However, there are several limitations that should be acknowledged. The study has a relatively low response rate of 20.6%, which means the results may not be representative of all Sydney gastroenterologists. Additionally, we only surveyed gastroenterologists based in one Australian metropolitan city (Sydney), which means the data should be interpreted as providing a local, rather than a nationally representative context.

Another limitation is the survey’s inability to address the physiological component of some defecation disorders, as bowel issues could be a symptom of underlying mental health problems such as depression and anxiety. Hence, future research should examine the role of clinical psychiatrists in the management of bowel issues. Finally, some questions in the survey could be interpreted differently by respondents. For example, when asked to “explain anorectal biofeedback procedures”, some respondents questioned to whom this definition would be given. This ambiguity might be avoided by providing additional detail to questions such as specifying the audience or context.

## 5. Conclusions

Although anorectal biofeedback is supported by clinical evidence and recommended by international guidelines, this scoping review and Australian cross-sectional study demonstrate that clinicians have limited knowledge of the therapy, and that access barriers majorly hinder its implementation. Improving clinician training, standardising treatment approaches, and addressing systemic obstacles are essential to improve the uptake of anorectal biofeedback in both global and local Australian settings. In conjunction with further research into biofeedback, these steps will ensure that patients with functional anorectal disorders receive optimal care through the translation of evidence-based recommendations into real-life clinical practice.

## Figures and Tables

**Table 1:** Summary of included studies.

First Author (Year)	Country	Aim	Sample Size (Response Rate)	Participants’ Occupation (Sampling Frame)	Participants’ Characteristics	Summary of Findings	Risk of Bias
Etherson, K.J. (2016) [16]	UK	To establish practitioners’ opinions on the technique, training, supervision, practice, efficacy, and experience of treating chronic idiopathic constipation using biofeedback therapy.	67 (unknown)	Nurses, physiotherapists, clinical physiologists (specialist national conferences on pelvic floor disorders, hospitals)	Majority (n = 48) had an NHS specified practice, with the rest being private (n = 3), NHS non-specific (n = 2), and foreign (n = 1).	Majority (63%) did not appropriately define biofeedback. Most common equipment used were digital/auditory (34%), visual (24%) and muscle stimulation (17%) devices. Most common methods used were lifestyle advice (98%), dietary advice, exercises, toileting position (97% for previous), urge resistance (83%), brace pump technique (78%), balloon catheter (54%), medication advice (12%) and transanal irrigation (6%). A significant proportion undertook informal training in biofeedback (95%) while majority underwent formal assessed training courses (65%). Informal training was delivered by peers (59%), mentors (46%) and manufacturers of biofeedback equipment (5%). Over half (52%) of those who did informal training were self-taught. The method included reading journal articles (63%), peer observation (47%), reading books (33%), attending conferences (17%) and study days (17%). Supervision of biofeedback was either formal (36%) or informal (64%) and mostly carried out by senior clinical colleagues or peers of similar grade. Supervision was either regular (41%), irregular (33%) or did not occur (26%). There were no statistically significant differences in the perceptions of biofeedback efficacy between nurses and physiotherapists. Perceptions differed from North to South UK, with higher efficacy reported in Southern UK (70% vs. 46%). Physiotherapists commonly indicated that they provide superior biofeedback therapy than nurse specialists. Barriers to use of biofeedback include long wait times, limited equipment and lack of personnel.	High
Al-Mozany, N. (2017) [17]	Australia, New Zealand	To identify the barriers to effective management of obstructed defecation (OD).	113 (68.50%)	Colorectal surgeons (Colorectal Surgical Society of Australia and New Zealand/CSSANZ)	Most (82%) aged between 40 and 60 years. Majority (87%) worked in a major metropolitan or university teaching hospital, with the rest in a provincial or rural centre. Most worked currently in Australia (81.5%) and the rest in New Zealand. Over half (67%) trained in Australia, with the remaining in New Zealand (12%) or elsewhere (11%), mainly the UK.	Anal manometry was reported to have a median importance of 4/5 in the management of OD. Majority (69%) had access to biofeedback, typically within a public hospital (35%). This was lower than access to a defecating proctogram (95%), anorectal physiologist (89%) and pelvic floor physiotherapist (92%).	Moderate
Mazor, Y. (2023) [18]	Australia, New Zealand	To compare the diagnostic and management approach for OD symptoms between gastroenterologists and colorectal surgeons.	107 (27.27%)	Colorectal surgeon (CSSANZ), gastroenterologists (Gastroenterological Society of Australia)	Gastroenterologists had a mean age of 52 years (SD 13 years), and colorectal surgeons had a mean age of 51 years (SD 9 years). Most common practice setting of gastroenterologists was public hospital inpatient (51%), followed by private outpatient clinic (38%), private hospital inpatient (27%), and public outpatient (1%). Colorectal surgeons mostly practised in public hospital inpatient (66%), followed by private hospital inpatient (44%), private outpatient clinic (13%), and public outpatient clinic (2%). Over half (51%) of gastroenterologists had 20 or more years of experience, 20% had 5–9 years of experience, 16% had 0–4 years of experience, and 13% had 10–19 years of experience. Colorectal surgeons most commonly had 10–19 years of experience (37%). The remaining surgeons had 20 or more (24%), 5–9 (24%), or 0–4 (15%) years of experience.	Minority of colorectal surgeons and gastroenterologists would use anorectal manometry and biofeedback to manage OD symptoms in various clinical scenarios. Majority (73% of gastroenterologists and 82% of colorectal surgeons) reported having access to anorectal manometry and biofeedback, less than access to a defecating proctogram.	Moderate
Losacco, L. (2022) [19]	Italy	To identify how pelvic floor diseases (PFD) are managed and the degree of integration between specialists.	37 (unknown)	General surgeons, gynaecologists, urologists (Italian Society of Colorectal Surgery)		Less than half reported the use of biofeedback, electrostimulation and physiokinesitherapy (45.9%) or biofeedback and electrostimulation (24.3%) to treat PFD. Majority (67%) reported use of anorectal manometry and/or urodynamic studies to diagnose PFD. Rehabilitative programs were available less than half of the time (48.6%) and always used in a minority of patients (13.5%). Most hospitals (86.5%) did not have a dedicated performance code for pelvic multidisciplinary examination.	High
Coppersmith, N.A. (2024) [20]	USA	To determine colorectal surgeons’ perceptions and practice patterns regarding low anterior resection syndrome (LARS) after rectal cancer treatment.	116 (30.10%)	Colorectal surgeons (Accreditation Council for Graduate Medical Education programs)	More males than females (55.2% vs. 40.5%). Majority (69%) worked at a university hospital. The remaining worked in a university-affiliated hospital (22.4%), independent or community teaching hospital (6%), private practice (1.7%), or independent or community hospital (0.9%). Highest proportion of colorectal surgeons had 11–20 years of experience (33.6%), followed by 20 or more (28.5%), 6–10 (15.5%), 3–5 (14.7%), and 0–2 (7.8%) years of experience.	Most common treatment for LARS after rectal cancer was lifestyle modifications with drugs (32.7%), followed by physical therapy (18.5%), lifestyle changes only (16.5%) and biofeedback (13.5%). Biofeedback was always used as treatment in 13.5% of cases, used sometimes in 38.5% of cases and never use in 6.7% of cases. Anorectal manometry and/or EMG were used routinely in 20.7% of patients with preoperative baseline incontinence/dysfunction and 22.4% of postoperative cases. As the number of years in practice increased, colorectal surgeons were less likely to use biofeedback (OR = 0.74, 95% CI = 0.54–1.00, *p* = 0.050).	Moderate
Gouriou, C. (2020) [21]	France	To investigate the therapeutic management of solitary rectal ulcer syndrome (SRUS).	91 (18.20%)	Colorectal surgeons, proctologists (French National Society of Coloproctology)	Mean age was 51.7 years (SD 11.1 years).	Majority (68%) reported that biofeedback should be proposed for treatment of SRUS before surgeries. There was no consensus on biofeedback use in surgical cases.	Moderate
Weinman, M.L. (1982) [22]	USA	To investigate physicians’ attitudes and knowledge of biofeedback therapy.	465 (13.50%)	Gastroenterologists, allergists, cardiologists, dermatologists, endocrinologists, internal medicine physicians, neurologists, obstetrician-gynaecologists, oncologists, ophthalmologists, paediatricians, psychiatrists, surgeons (Harris County Medical Society)		Most had little knowledge about biofeedback (62%), with 23% having moderate knowledge and 11.6% having significant knowledge. Neurologists, psychiatrists and allergists had higher levels of knowledge than other physicians. 42.3% had no opinion on the use of biofeedback in the treatment of faecal incontinence. Minority (4.2%) reported using biofeedback as the primary treatment for faecal incontinence. 22.4% would use biofeedback as an adjunct treatment and 23.2% believed biofeedback was not indicated. Most (86.2%) reported not using biofeedback. 21.7% were willing to refer patients for biofeedback. 27% reported health insurance coverage for biofeedback, 16.9% reported no coverage and 47.1% were undecided about this.	High
Jimenez-Gomez, L.M. (2016) [23]	Spain	To determine surgeons’ knowledge, opinions, assessment, and treatment of LARS.	484 (5.80%)	Colorectal surgeons, proctologists (American Society of Colon and Rectal Surgeons, Spanish Association of Surgeons, Spanish Society of Coloproctology)	Spanish surgeons most commonly worked at a university hospital with more than 600 beds (36%), followed by university hospital with 400–600 beds (21%), public hospital not affiliated with a university hospital (19%), university hospital with less than 400 beds (15%), and private centres (8%). American surgeons most commonly worked at a private centre (34%), followed by university hospital with over 600 beds (28%), university hospital with 400–600 beds (22%), public hospital not affiliated with university (9%), and university hospital with less than 400 beds (8%). Almost half worked in USA (45.5%), with the rest working in Canada, Brazil, Japan, UK, Italy, Australia, Korea, Greece, and Turkey among other countries.	8.3% of American surgeons and 10.0% of Spanish surgeons preferred treating LARS with biofeedback. Less surgeons chose biofeedback over lifestyle and dietary modification with or without drugs. More American surgeons chose drugs only over biofeedback whereas the opposite was seen in Spanish surgeons. Over half (53.1% of American surgeons and 57.3% of Spanish surgeons) reported biofeedback was useful for treatment of LARS. 14.2% of American surgeons and 23.3% of Spanish surgeons reported biofeedback was the most effective treatment of defecatory functional impairment after LARS. 55.6% of American surgeons and 40.7% of Spanish surgeons only had experience in drug and dietary treatment.	Moderate

**Table 2:** Characteristics of gastroenterologists included in this study (n = 46).

Characteristics	Number of Gastroenterologists, n (%)
Gender	
Male	31 (67.4%)
Female	15 (32.6%)
Type of practice	
Public	7 (15.2%)
Private	6 (13.0%)
Both	33 (71.7%)
Local Health District	
Sydney	14 (19.7%)
South Western Sydney	12 (16.9%)
South Eastern Sydney	9 (12.7%)
Northern Sydney	8 (11.3%)
Nepean Blue Mountains	6 (8.5%)
Western Sydney	4 (8.5%)
Central Coast	4 (5.6%)
Illawarra Shoalhaven	3 (4.2%)
Murrumbidgee	3 (4.2%)
Mid North Coast	2 (2.8%)
Hunter New England	1 (1.4%)
Other	3 (4.2%)

**Table 3:** Methods to improve usage of anorectal biofeedback therapy.

Methods	Number of Gastroenterologists, n (%)
Education of gastroenterologists	25 (20.3%)
Wider access	23 (18.7%)
Government subsidies	15 (12.2%)
Education of general practitioners	14 (11.4%)
Government funding of facilities	11 (8.9%)
More multidisciplinary interactions	10 (8.1%)
Education of medical students	7 (5.7%)
Education of colorectal/general surgeons	7 (5.7%)
Patient compliance	4 (3.3%)
Education of public	3 (2.4%)
Education of specialist nurses	2 (1.6%)
Education of physiotherapists/exercise physiologists	1 (0.8%)
More research	1 (0.8%)

**Table 4:** Reasons why anorectal biofeedback is not offered at clinic or hospital (n = 31).

Reasons	Number of Gastroenterologists, n (%)
No one qualified	12 (38.7%)
Costs	8 (25.8%)
Lack of knowledge	6 (19.4%)
Unsuited to patient	2 (6.5%)
Geographical challenge	1 (3.2%)
Lack of access	1 (3.2%)
Not priority	1 (3.2%)

**Table 5:** Conditions treated by anorectal biofeedback therapy.

Conditions	Number of Gastroenterologists, n (%)
Faecal incontinence	19 (19.2%)
Dyssynergic defecation	18 (19.2%)
Chronic constipation	17 (17.2%)
Anorectal dyssynergia	17 (17.2%)
Pelvic floor dysfunction	13 (13.1%)
Anal pain	7 (7.0%)
Post-surgical rehabilitation	4 (4.0%)
Tenesmus	4 (4.0%)

**Table 6:** Components of anorectal biofeedback therapy.

Components	Number of Gastroenterologists n, (%)
Lifestyle advice	17 (16.8%)
Toileting position	15 (14.9%)
Dietary advice	14 (13.9%)
Medications advice	13 (12.9%)
Exercises	12 (11.9%)
Manometry	9 (8.9%)
Balloon catheter	8 (7.9%)
Counselling	5 (5.0%)
Unsure as referred	4 (4.0%)
Transanal irrigation	3 (3.0%)
Brace pump technique	1 (1.0%)

## Data Availability

The raw data supporting the conclusions of this article will be made available by the authors on request.

## Supplementary Materials

The following supporting information can be downloaded at: https://www.mdpi.com/article/10.3390/jcm15020835/s1, Table S1: Preferred Reporting Items for Systematic reviews and Meta-Analyses extension for Scoping Reviews (PRISMA-ScR) Checklist [37]; Supplementary Materials S2: Survey Questions.

## Appendix A

MEDLINE: ((exp nurses/or gastroenterologists/or general practitioners/or physicians, primary care/or physicians, family/or surgeons/or physical therapists/) or (gastroenterologist* or ‘colorectal surgeon*’ or ‘general practitioner*’ or physiotherapist* or nurse* or ‘physical therapist*’ or physician* or doctor* or clinician*).ti,ab.) and (exp biofeedback, psychology/or biofeedback.ti,ab.) and ((exp constipation/or exp defecation/or exp anus diseases/or exp fecal incontinence/or exp low anterior resection syndrome/or exp rectal prolapse/or exp rectocele/or exp pain, postoperative/) or (‘f*ecal incontinence’ or ‘obstructed def*ecation’ or ‘dyssynergic defecation’ or constipation or anismus or ‘pelvic floor dyssynergia’ or ‘pelvic floor dysfunction’ or ‘levator ani syndrome’ or ‘lower anterior resection syndrome’ or ‘rectal prolapse’ or rectocele or ‘anal fissure’ or ‘anorectal pain’ or ‘post-operative pain’ or ‘post-surgical rehabilitation’).ti,ab.) and ((exp ‘attitude of health personnel’/or health knowledge, attitudes, practice/) or (attitude* or perception* or perceived or perspective* or opinion* or knowledge or survey* or experience* or understanding).ti,ab.)

Embase: ((exp nurse/or gastroenterologist/or general practitioner/or surgeon/or exp physiotherapist/) or (gastroenterologist* or ‘colorectal surgeon*’ or ‘general practitioner*’ or physiotherapist* or nurse* or ‘physical therapist*’ or physician* or doctor* or clinician*).ti,ab.) and (biofeedback/or biofeedback.ti,ab.) and ((constipation/or exp chronic constipation/or defecation disorder/or anorectal disease/or exp anus disease/or exp rectum disease/or exp feces incontinence/or exp low anterior resection syndrome/or exp rectum prolapse/or exp rectocele/or pelvic floor disorder/or exp postoperative pain/) or (‘f*ecal incontinence’ or ‘obstructed def*ecation’ or c2onstipation or anismus or ‘pelvic floor dyssynergia’ or ‘pelvic floor dysfunction’ or ‘levator ani syndrome’ or ‘lower anterior resection syndrome’ or ‘rectal prolapse’ or rectocele or ‘anal fissure’ or ‘anorectal pain’ or ‘post-operative pain’ or ‘post-surgical rehabilitation’).ti,ab.) and ((health personnel attitude/or physician attitude/or physiotherapist attitude/or professional knowledge/) or (attitude* or perception* or perceived or perspective* or opinion* or knowledge or survey* or experience* or understanding).ti,ab.)

CINAHL: (((MH ‘Physical Therapists’) or (MH ‘Nurses’) or (MH ‘Gastroenterologists’) or (MH ‘Physicians, Family’) or (MH ‘Surgeons’)) or (TI(gastroenterologist* or ‘colorectal surgeon*’ or ‘general practitioner*’ or physiotherapist* or nurse* or ‘physical therapist*’ or physician* or doctor* or clinician*) or AB(gastroenterologist* or ‘colorectal surgeon*’ or ‘general practitioner*’ or physiotherapist* or nurse* or ‘physical therapist*’ or physician* or doctor* or clinician*))) and ((MH ‘Biofeedback’) or (TI(biofeedback) or AB(biofeedback))) and (((MH ‘Constipation’) or (MH ‘Fecal Incontinence’) or (MH ‘Rectal Prolapse’) or (MH ‘Rectocele’) or (MH ‘Lower Anterior Resection Syndrome’) or (MH ‘Pelvic Floor Disorders’) or (MH ‘Postoperative Pain’) or (MH ‘Fissure in Ano’)) or (TI(‘f*ecal incontinence’ or ‘obstructed def*ecation’ or constipation or anismus or ‘pelvic floor dyssynergia’ or ‘pelvic floor dysfunction’ or ‘levator ani syndrome’ or ‘lower anterior resection syndrome’ or ‘rectal prolapse’ or rectocele or ‘anal fissure’ or ‘anorectal pain’ or ‘post-operative pain’ or ‘post-surgical rehabilitation’) or AB(‘f*ecal incontinence’ or ‘obstructed def*ecation’ or constipation or anismus or ‘pelvic floor dyssynergia’ or ‘pelvic floor dysfunction’ or ‘levator ani syndrome’ or ‘lower anterior resection syndrome’ or ‘rectal prolapse’ or rectocele or ‘anal fissure’ or ‘anorectal pain’ or ‘post-operative pain’ or ‘post-surgical rehabilitation’))) and (((MH ‘Physician Attitudes’) or (MH ‘Nurse Attitudes’) or (MH ‘Physical Therapist Attitudes’) or (MH ‘Professional Knowledge+’)) or (TI(attitude* or perception* or perceived or perspective* or opinion* or knowledge or survey* or experience* or understanding) or AB(attitude* or perception* or perceived or perspective* or opinion* or knowledge or survey* or experience* or understanding)))

Scopus: (TITLE (gastroenterologist* or ‘colorectal surgeon*’ or ‘general practitioner*’ or physiotherapist* or nurse* or ‘physical therapist*’ or physician* or doctor* or clinician*) or ABS (gastroenterologist* or ‘colorectal surgeon*’ or ‘general practitioner*’ or physiotherapist* or nurse* or ‘physical therapist*’ or physician* or doctor* or clinician*)) and (TITLE (biofeedback) or ABS (biofeedback)) and (TITLE (‘f*ecal incontinence’ or ‘obstructed def*ecation’ or ‘dyssynergic defecation’ or constipation or anismus or ‘pelvic floor dyssynergia’ or ‘pelvic floor dysfunction’ or ‘levator ani syndrome’ or ‘lower anterior resection syndrome’ or ‘rectal prolapse’ or rectocele or ‘anal fissure’ or ‘anorectal pain’ or ‘post-operative pain’ or ‘post-surgical rehabilitation’) or ABS (‘f*ecal incontinence’ or ‘obstructed def*ecation’ or ‘dyssynergic defecation’ or constipation or anismus or ‘pelvic floor dyssynergia’ or ‘pelvic floor dysfunction’ or ‘levator ani syndrome’ or ‘lower anterior resection syndrome’ or ‘rectal prolapse’ or rectocele or ‘anal fissure’ or ‘anorectal pain’ or ‘post-operative pain’ or ‘post-surgical rehabilitation’)) and (TITLE (attitude* or perception* or perceived or perspective* or opinion* or knowledge or survey* or experience* or understanding) or ABS (attitude* or perception* or perceived or perspective* or opinion* or knowledge or survey* or experience* or understanding))

APA PsycInfo: (((DE ‘Physical Therapists’) or (DE ‘Family Physicians’) or (DE ‘General Practitioners’) or (DE ‘Surgeons’) or (DE ‘Nurses’)) or (TI ( gastroenterologist* or ‘colorectal surgeon*’ or ‘general practitioner*’ or physiotherapist* or nurse* or ‘physical therapist*’ or physician* or doctor* or clinician* ) or AB ( gastroenterologist* or ‘colorectal surgeon*’ or ‘general practitioner*’ or physiotherapist* or nurse* or ‘physical therapist*’ or physician* or doctor* or clinician* ))) and (DE ‘Biofeedback’ or (TI biofeedback or AB biofeedback)) and (((DE ‘Constipation’) or (DE ‘Fecal Incontinence’) or (DE ‘Defecation’)) or (TI ( ‘f*ecal incontinence’ or ‘obstructed def*ecation’ or ‘dyssynergic defecation’ or constipation or anismus or ‘pelvic floor dyssynergia’ or ‘pelvic floor dysfunction’ or ‘levator ani syndrome’ or ‘lower anterior resection syndrome’ or ‘rectal prolapse’ or rectocele or ‘anal fissure’ or ‘anorectal pain’ or ‘post-operative pain’ or ‘post-surgical rehabilitation’ ) or AB ( ‘f*ecal incontinence’ or ‘obstructed def*ecation’ or ‘dyssynergic defecation’ or constipation or anismus or ‘pelvic floor dyssynergia’ or ‘pelvic floor dysfunction’ or ‘levator ani syndrome’ or ‘lower anterior resection syndrome’ or ‘rectal prolapse’ or rectocele or ‘anal fissure’ or ‘anorectal pain’ or ‘post-operative pain’ or ‘post-surgical rehabilitation’ ))) and (((DE ‘Health Attitudes’) or (DE ‘Health Knowledge’)) or (TI ( attitude* or perception* or perceived or perspective* or opinion* or knowledge or survey* or experience* or understanding ) or AB ( attitude* or perception* or perceived or perspective* or opinion* or knowledge or survey* or experience* or understanding )))

## Appendix B

	**Yes**	**No**	**Unclear**	**Not Applicable**
1. Were the criteria for inclusion in the sample clearly defined?	□	□	□	□
2. Were the study subjects and the setting described in detail?	□	□	□	□
3. Was the exposure measured in a valid and reliable way?	□	□	□	□
4. Were objective, standard criteria used for measurement of the condition?	□	□	□	□
5. Were confounding factors identified?	□	□	□	□
6. Were strategies to deal with confounding factors stated?	□	□	□	□
7. Were the outcomes measured in a valid and reliable way?	□	□	□	□
8. Was appropriate statistical analysis used?	□	□	□	□
Overall appraisal: Include □; Exclude □; Seek further info □.				
