# Supplementary material for: A Global Scoping Review of Clinicians’ Perceptions of Anorectal Biofeedback Compared with Novel Australian Data
Source: J Clin Med. 2026 Jan 20;15(2):835. doi: 10.3390/jcm15020835 (PMC12842376; doi:10.3390/jcm15020835)
Supplement: Supplementary file 1 [file jcm-15-00835-s001.zip › S2.pdf]

Anorectal Biofeedback Research Study

Information

0. This survey aims to understand the perceptions of gastroenterologists like yourself regarding anorectal biofeedback therapy in Australia. Despite its proven efficacy in treating conditions like faecal incontinence and chronic constipation, the uptake of anorectal biofeedback therapy in Australia is remarkably low (less than 1% of patients with bowel or bladder control problems). This survey is part of a research study conducted by researchers at Western Sydney University and will only take 2-3 minutes to complete. Participation is entirely voluntary, and your responses will be kept anonymous and confidential. The data collected will be used solely for academic research purposes and may be used in future research projects. Thank you in advance for your valuable insight!

Please click 'Yes' if you would like to proceed and 'No' if you would like to opt out.

- ○ Yes
- ○ No

Eligibility

1. Did you undertake gastroenterology specialty training in Australia?

- ○ Yes
- ○ No

2. Have you worked in Australia for the past 3 years?

- ○ Yes
- ○ No

Demographics

3. What is your gender?

- ○ Male
- ○ Female
- ○ [ ] Other (specify)

4. How many years have you been practising in gastroenterology? (Enter a number - e.g. 5)

5. Do you work in a public or private hospital/clinic?

- ○ Public
- ○ Private
- ○ Both

6. Which Local Health District(s) (LHD) in NSW have you worked in? If you practise privately, choose LHDs that correspond to areas that you have worked in (You can choose more than one option)

- ☐ Central Coast
- ☐ Far West
- ☐ Hunter New England
- ☐ Illawarra Shoalhaven
- ☐ Mid North Coast
- ☐ Murrumbidgee
- ☐ Nepean Blue Mountains
- ☐ Northern NSW
- ☐ Northern Sydney
- ☐ South Eastern Sydney
- ☐ South Western Sydney
- ☐ Southern NSW
- ☐ Sydney

☐ Western NSW

☐ Western Sydney

☐ [ ] Other (e.g. interstate, international)

Knowledge About Biofeedback

7. How many patients with bowel symptoms relating to anal dysfunction (e.g. chronic constipation, faecal incontinence) do you see each week on average? (Enter a number - e.g. 10)

[ ]

8. Are you aware of anorectal biofeedback?

○ Yes

○ No

Anorectal Biofeedback

9. How would you explain anorectal biofeedback procedures in 1-2 sentences?

10. Is anorectal biofeedback offered as a treatment option at your clinic/hospital? (This includes referrals to other facilities)

- ○ Yes
- ○ No

11. Why is anorectal biofeedback not offered as a treatment option at your clinic/hospital?

- ○ Unsuited to patient
- ○ Geographical challenge to patients
- ○ No one qualified to deliver it
- ○ Lack of knowledge about it
- ○ Financial costs
- ○ Other (specify)

11. How often do you recommend anorectal biofeedback therapy to your patients? Enter '[number] times weekly/monthly/yearly' (e.g. 3 times monthly)

12. What percentage of these patients benefit from biofeedback therapy? A rough estimate is sufficient (e.g. 40%)

13. How do you determine the effectiveness of biofeedback therapy?

- ○ Patient feedback
- ○ Nurse/physiotherapist feedback
- ○ Objective parameters
- ○ Other (specify)

14. What conditions do you recommend anorectal biofeedback therapy for? (You can choose more than one)

- ☐ Faecal incontinence
- ☐ Chronic constipation
- ☐ Pelvic floor dysfunction

- ☐ Anorectal dyssynergia
- ☐ Post-surgical rehabilitation
- ☐ Dyssynergic defecation
- ☐ Anal pain
- ☐ Tenesmus
- ☐ [ ] Other (specify)

15. Which of these components are integrated into your biofeedback therapy program? (You can choose more than one)

- ☐ Lifestyle advice
- ☐ Dietary advice
- ☐ Medication advice
- ☐ Toileting position
- ☐ Exercises
- ☐ Balloon catheter
- ☐ Manometry based testing
- ☐ Brace pump technique
- ☐ Transanal irrigation
- ☐ Psychological counselling
- ☐ [ ] Other (specify)

16. What equipment is used in your anorectal biofeedback therapy program? (You can choose more

than one)

- Auditory devices
- Visual devices
- Muscle stimulation devices
- Not sure
- [ ] Other (specify)

Attitudes Towards Biofeedback

17. How effective do you believe anorectal biofeedback is as a therapy for patients?

	1	2	3	3	4	5
Scale of 1 to 5 (1=not effective at all to 5=extremely effective)						

18. Why or why not is anorectal biofeedback effective? (Please be as specific as possible)

19. What are the 3 most effective ways to improve usage of anorectal biofeedback therapy? (Pick up to 3 options)

- ☐ Education of medical students
- ☐ Education of general practitioners (GPs)
- ☐ Education of gastroenterologists
- ☐ Education of colorectal/general surgeons
- ☐ Education of specialist nurses
- ☐ Education of physiotherapists/exercise physiologists
- ☐ Education of general public
- ☐ Government funding of anorectal biofeedback facilities
- ☐ Government subsidies for anorectal biofeedback therapy (e.g. Medicare number)
- ☐ Wider access to anorectal biofeedback (e.g. more clinics/services)
- ☐ More research into anorectal biofeedback
- ☐ More multidisciplinary interactions about anorectal biofeedback
- ☐ Patient compliance with the procedure(s)
- ☐ [ ] Other (specify)

20. Do you have any additional comments/opinions on anorectal biofeedback?

[ ]

Anorectal Conditions

9. Are you comfortable managing most cases of faecal incontinence?

- ○ Yes
- ○ No

10. What treatments do you recommend for faecal incontinence? (You can choose more than one)

- ☐ Dietary advice
- ☐ Lifestyle advice
- ☐ Toileting position advice
- ☐ Enema or suppositories
- ☐ Oral laxatives
- ☐ Retrograde or antegrade irrigation
- ☐ Antidiarrhoeal medication
- ☐ Neosphincter surgery
- ☐ Stoma surgery
- ☐ Pelvic floor exercises
- ☐ Bulking agents
- ☐ Electrical nerve stimulation
- ☐ ________ Other (specify)

11. Are you comfortable managing most cases of chronic constipation?

- ○ Yes
- ○ No

12. What treatments do you recommend for chronic constipation?

- ☐ Osmotic or stimulant laxatives
- ☐ Bulk or fruit-based laxatives
- ☐ Dietary advice
- ☐ Lifestyle advice
- ☐ Toileting position advice
- ☐ Pro-kinetic medications
- ☐ Retrograde or antegrade irrigation
- ☐ Electrical stimulation
- ☐ Enemas or suppositories
- ☐ Withdrawal of medication
- ☐ Psychological counselling
- ☐ Antidepressants
- ☐ Surgery
- ☐ [ ] Other

Attitudes Towards Biofeedback 2

13. How effective do you believe anorectal biofeedback is as a therapy for patients?

	1	2	3	3	4	5
Scale of 1 to 5 (1=not effective at all to 5=extremely effective)	○					

14. Why or why not is anorectal biofeedback effective? (Please be as detailed as possible)

15. What are ways to improve usage of anorectal biofeedback therapy? (You can pick more than one)

- ☐ Education of medical students
- ☐ Education of general practitioners (GPs)
- ☐ Education of gastroenterologists
- ☐ Education of colorectal/general surgeons
- ☐ Education of physiotherapists/exercise physiologists
- ☐ Education of general public
- ☐ Government funding of anorectal biofeedback facilities

- [ ] Government subsidies for anorectal biofeedback therapy (e.g. Medicare number)
- [ ] Wider access to anorectal biofeedback (e.g. more clinics/services)
- [ ] More research into anorectal biofeedback
- [ ] More multidisciplinary interactions about anorectal biofeedback
- [ ] Other (specify) ______

16. Do you have any additional comments/opinions on anorectal biofeedback?

______

We really appreciate your insights! Please click 'Next page' to continue :)

Powered by Qualtrics
